# Changes in the gene expression programs of renal mesangial cells during diabetic nephropathy

**DOI:** 10.1186/1471-2369-13-70

**Published:** 2012-07-28

**Authors:** Eric W Brunskill, S Steven Potter

**Affiliations:** 1 Division of Developmental Biology, Children’s Hospital Medical Center, Cincinnati, Ohio, 45229, USA

**Keywords:** Mesangial cells, Diabetic nephropathy, Fibrosis

## Abstract

**Background:**

Diabetic nephropathy is the leading cause of end stage renal disease. All three cell types of the glomerulus, podocytes, endothelial cells and mesangial cells, play important roles in diabetic nephropathy. In this report we used *Meis1-GFP* transgenic mice to purify mesangial cells from normal mice and from *db*/*db* mice, which suffer diabetic nephropathy. The purpose of the study is to better define the unique character of normal mesangial cells, and to characterize their pathogenic and protective responses during diabetic nephropathy.

**Methods:**

Comprehensive gene expression states of the normal and diseased mesangial cells were defined with microarrays. By comparing the gene expression profiles of mesangial cells with those of multiple other renal cell types, including podocytes, endothelial cells and renal vesicles, it was possible to better define their exceptional nature, which includes smooth muscle, phagocytic and neuronal traits.

**Results:**

The complete set of mesangial cell expressed transcription factors, growth factors and receptors were identified. In addition, the analysis of the mesangial cells from diabetic nephropathy mice characterized their changes in gene expression. Molecular functions and biological processes specific to diseased mesangial cells were characterized, identifying genes involved in extracellular matrix, cell division, vasculogenesis, and growth factor modulation. Selected gene changes considered of particular importance to the disease process were validated and localized within the glomuerulus by immunostaining. For example, thrombospondin, a key mediator of TGFβ signaling, was upregulated in the diabetic nephropathy mesangial cells, likely contributing to fibrosis. On the other hand the *decorin* gene was also upregulated, and expression of this gene has been strongly implicated in the reduction of TGFβ induced fibrosis.

**Conclusions:**

The results provide an important complement to previous studies examining mesangial cells grown in culture. The remarkable qualities of the mesangial cell are more fully defined in both the normal and diabetic nephropathy diseased state. New gene expression changes and biological pathways are discovered, yielding a deeper understanding of the diabetic nephropathy pathogenic process, and identifying candidate targets for the development of novel therapies.

## Background

The glomerulus is composed of three main cell types, podocytes, endothelial cells and mesangial cells. Mesangial cells provide the extracellular matrix framework upon which the glomerulus is built. In addition they have smooth muscle-like properties and are able to regulate capillary flow. Mesangial cells also have a phagocytic function, and are capable of carrying out disposal and degradation of filtrate debris.

Mesangial cells play important roles in many kidney diseases, including diabetic nephropathy. Type2 diabetes is showing increasing global prevalence and is now the most common cause of end stage renal disease [1]. All three major cell types of the glomerulus have been implicated in diabetic nephropathy. The mesangial cells are responsible for the observed accumulation of extracellular matrix and mesangial expansion [2].

Because of their remarkable properties mesangial cells have been the subject of intense investigation. In particular, many studies have used microarrays to better understand these cells. In most of these studies mesangial cells were grown in culture and exposed to treatments relevant to various disease conditions, and the resulting changes in gene expression profiles were determined with microarrays. Treatments included high glucose [3], static pressure [4], serum [5], kynurenine metabolites [6], endothelin [7] and PDGF [8]. The results defined the multiple gene expression responses of mesangial cells to these stimuli, and thereby provided insight into the molecular mechanisms of mesangial cell mediated pathogenesis. These studies were limited, however, by the use of mesangial cells in culture, which cannot fully replicate the *in vivo* cell, and by the restricted sets of treatments, which cannot completely reproduce the complex micro-environmental changes experienced by mesangial cells during diabetic nephropathy.

In this report we define *in vivo* mesangial cell gene expression programs during health and disease. The *Meis1-GFP* transgene specifically marks mesangial cells within the glomerulus, allowing their purification through fluorescence activated cell sorting (FACS), and subsequent gene expression profiling with microarrays. By comparing the mesangial gene expression pattern to those of other renal cell types it was possible to begin to define the unique character of these remarkable cells. In addition we purified mesangial cells from *db*/*db* mutant mice with diabetic nephropathy, allowing the determination of both pathogenic and protective changes in gene expression as a function of disease.

## Methods

### Purification of mesangial cells

*Meis1-GFP* transgenic mice, *Tg(Meis1-EGFP)FO156Gsat*, were obtained from GENSAT/MMRC (http://www.gensat.org/MMRRC_report.jsp). The *Meis1-GFP* marks both stromal (interstitial) cells and mesangial cells, so it was essential to first purify glomeruli, to remove stroma. Glomeruli were isolated by sieving, a single cell suspension made, and mesangial cells FACS sorted as previously described [9]. The *db/db Meis1-GFP* mice were made by crossing *db*/+ and *db*/+ *Meis1-GFP* mice, with one eighth of progeny giving the desired genotype.

This study was carried out in strict accordance with the recommendations of the Guide for the Care and Use of Laboratory Animals of the National Institutes of Health. The protocol was approved by the Cincinnati Children's Research Foundation Institutional Animal Care and Use Committee (protocol number 0D02013).

### Purity

The microarray data was analyzed to insure the purity of the mesangial cells. As described in Results we screened for podocyte specific genes and found several that showed low, near background, expression in the mesangial cell profiles, arguing for very limited podocyte contamination. A similar analysis of genes associated with endothelial cells was performed giving similar results. The endothelial specific markers *Pecam1, KDR, Tspan8, Plac8* and *Eltd1* all showed strong expression in endothelial cells, as expected, and very low expression in the mesangial cells. It is interesting to note, however, that some endothelial specific marker genes did show significant expression in the mesangial cells. For example *Tek*, also known as *Tie2*, showed expression levels of about 3,000 in the glomerular endothelial cells, but also a considerable expression level of about 1,000 in mesangial cells. We argue that this is not the result of contamination since other endothelial cell markers showed low expression in the mesangial cells, but rather reflected a partial endothelial phenotype for the mesangial cells. We also examined the expression data for the presence of marker genes from other compartments of the kidney. For example *Ace, Acsm1Aqp11Slc18a1Slc22a6* and *Spp2* all should show restricted expression in the proximal tubule of the kidney [10], and each showed very low expression levels in the mesangial cells.

### RNA purification and Target Amplification

RNA was purified using Qiagen RNeasy Micro Kits. Target amplifications were carried out using RiboSpia technology from Nugen, with the Ovation Pico WTA system. Affymetrix standard methods were used for carrying out microarray hybridizations, washes and scans.

### Array data analysis

Data was analyzed primarily using Agilent GeneSpring 11.5.1 software. Specific parameters are described in Results. In addition we used ToppGene (http://toppgene.cchmc.org/) [11], ToppCluster (http://toppcluster.cchmc.org/) [12] and Cytoscape (http://www.cytoscape.org/) [13] for functional analysis and preparation of figures.

Microarray data is available on the public resource GUDMAP (www.gudmap.org).

## Results and discussion

### The unique gene expression character of the mesangial cell

Mice with the BAC *Meis1-GFP* transgene show specific GFP labeling of both stromal and mesangial cells in the kidney [10]. It was therefore possible to isolate a very pure population of mesangial cells from adult mouse kidneys by first purifying the glomeruli, using a sieving procedure, followed by preparation of a single cell suspension and FACS sorting of the GFP positive mesangial cells. Samples were prepared in biological triplicate, and gene expression profiles defined using Affymetrix Mouse Gene 1.0 ST microarrays.

**Figure 1:**
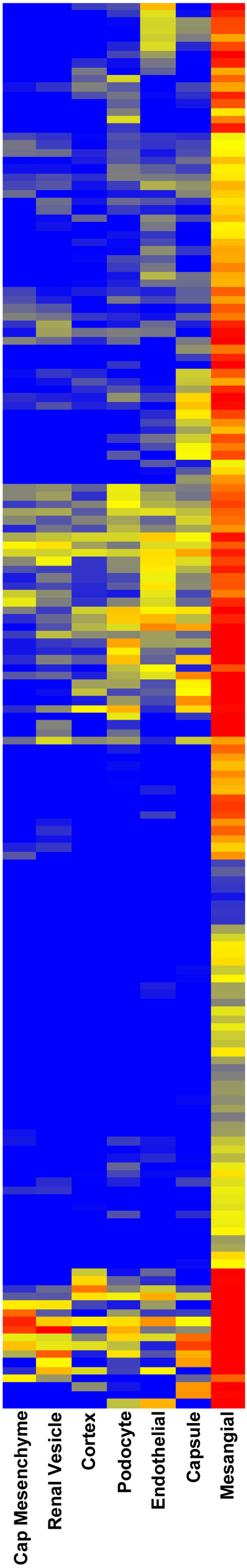
**Heatmap showing genes with elevated expression in normal mesangial cells.** Expression patterns of 172 genes with up-regulation in mesangial cells are compared across multiple renal compartments, including cap mesenchyme, renal vesicle, total renal cortex, podocytes, endothelial cells and renal capsule. Red indicates high, yellow represents intermediate and blue indicates low gene expression level. Most of the 172 genes are not uniquely expressed in mesangial cells, but do show significantly elevated mesangial expression. For an image that can be zoomed to visualize gene names see Additional file 2: Figure S1.

**Figure 2:**
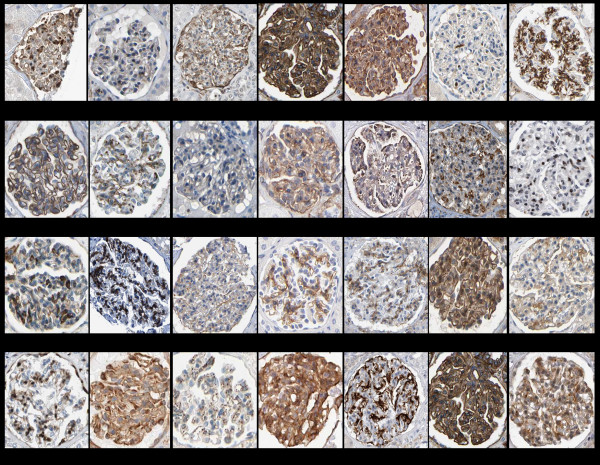
**Immunohistochemistry showing glomerular expression patterns of some of the genes with elevated mesangial cell expression.** The proteins of some genes, such as *Nr2f1*, show nuclear localization, as would be expected for a transcription factor, while in other cases, such as for PREX2, there is a striking extracellular localization. Data is from the human protein atlas (http://www.proteinatlas.org/).

To better understand the unique character of mesangial cells we contrasted their gene expression patterns to those of other cell types, including podocytes, endothelial cells, kidney capsule, total kidney cortex, renal vesicles, and cap mesenchyme. The end result was a list of 172 genes with distinctively elevated expression in mesangial cells (Additional file 1: Table S1). A heat map showing the relative expression levels of these genes is shown in Figure 1. (See Additional file 2: Figure S1 for an expandable heat map with genes named). We used the human protein atlas (http://www.proteinatlas.org/) to confirm expression of twenty eight genes predicted by the microarray data to have elevated mesangial cell expression (Figure 2).

To begin to determine the molecular functions and biological processes associated with these genes we used ToppGene (http://toppgene.cchmc.org/). Some of the strongest statistical hits for biological processes, with P-values of essentially zero, were found for wound healing, coagulation, locomotion, regulation of body fluid levels, and muscle contraction. Other biological processes of interest included inflammatory processes, ossification, cell adhesion, and extracellular matrix organization. Quite striking, there was also a gene expression signature of neural character, including behavior, cognition, learning, axogenesis, and neuron development. The picture that emerges describes a remarkable cell type with a markedly diverse set of biological functions. For complete lists see Additional file 3: Table S2.

**Figure 3:**
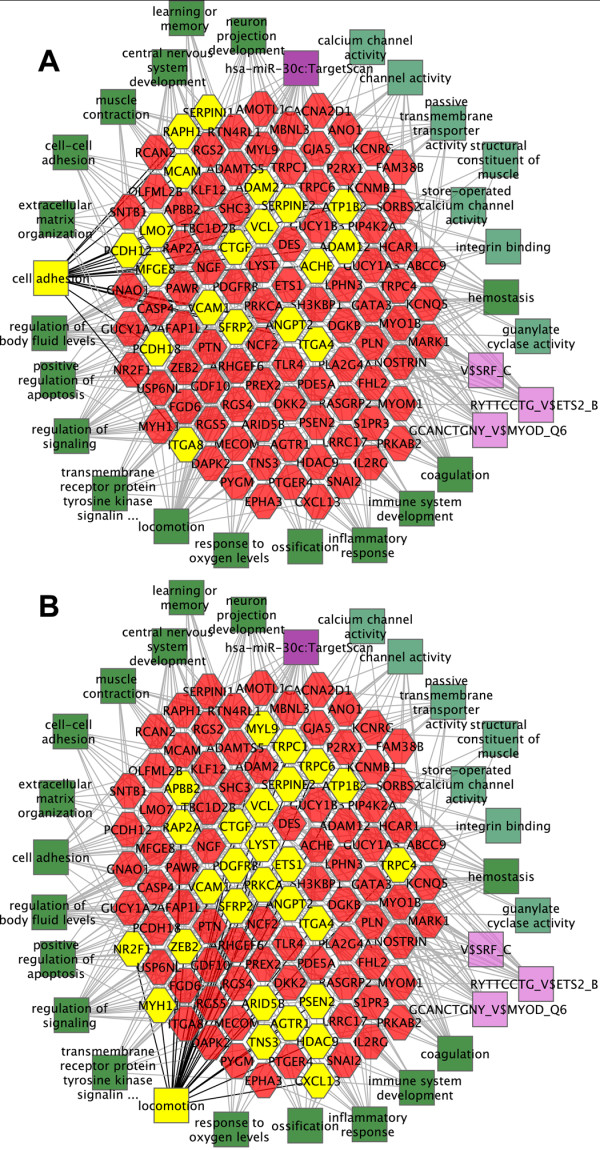
**Molecular function, biological processes and candidate regulatory transcription factors and microRNAs in normal mesangial cells.** The central hexagons show some of the 172 genes with elevated expression in normal mesangial cells, compared to other cell types. The surrounding squares show color coded molecular functions (light green), biological processes (dark green), transcription factors (light purple) and microRNAs (dark purple), strongly associated with these genes. Lines show connections to linked genes. **A**. The cell adhesion biological process, with associated genes, is highlighted in yellow. **B**. The locomotion biological process and associated genes is highlighted in yellow. For complete lists of molecular functions, biological processes, transcription factors, microRNAs and other features see Additional file 3: Table S2.

The results can be presented diagrammatically as cytoscapes (Figure 3). The center hexagons represent some of the genes with mesangial elevated expression, and the surrounding squares are color coded to show molecular functions, biological processes, transcription factors and microRNAs strongly associated with the connected genes.

### Transcription factors

Transcription factors play a key role in determining cell identity. The screen identified fourteen transcription factor genes with significantly elevated expression in mesangial cells, including *Gata3*, *Mecom*, *Pawr*, *Arid5b*, *Zeb2*, *Ets1*, *Apbb2*, *Snai2*, *Klf12, Hopx*, *Hdac9, Nr2f1*, *Ebf1* and *Rybp*, as ranked according to expression level.

It has been previously shown that *Gata3* is selectively expressed in the ureteric bud as well as mesangial cells and their adjacent endothelial cells during kidney development. *GATA3* haploinsufficiency in humans causes hypoparathryoidism, sensineural deafness and renal anomalies, including aplasia, dysplasia and hypoplasia [14].

*Mecom*, also known as *Evi1*, encodes a zinc finger transcription factor. It is intriguing to note that MECOM has been shown to physically interact with GATA protein, and it has also been shown to inhibit induction of some TGFβ target genes, and to play roles in cell proliferation and apoptosis [15].

The PAWR leucine zipper protein was shown to sensitize cells to apoptotic signals and was originally identified as a gene induced in apoptotic prostate cancer cells [16]. Of interest, given mesangial cell function in the synthesis of extracellular matrix, PAWR has also been shown to regulate secretase cleavage of amyloid precursor protein, and has thereby been implicated in Alzheimer disease [17]. PAWR was also shown to interact with WT1 and to enhance its transcriptional repression activity [18]. This might seem irrelevant since *Wt1* is generally though to be associated with podocytes, and not mesangial cells. We observed very robust expression of *Pawr* in mesangial cells, and about a three fold lower level in podocytes. Of considerable interest, however, we also observed an inverse gene expression pattern for *Wt1*, with raw signal expression in podocytes of about 3400, and a lower but still strong expression level of about 650 in mesangial cells.

This surprisingly suggests that *Wt1* is expressed in mesangial cells as well as podocytes. Could this be due to contamination of the mesangial cells with podocytes? We tested the data for possible contamination by examining the mesangial gene expression profile for other genes thought to be podocyte specific. If they all showed significant expression in the putative mesangial cells then this would argue strongly in favor of contamination. If, on the other hand, some are indeed truly podocyte specific in the dataset then this would rule out contamination as an explanation. It is difficult to envisage a scenario where the transcripts of some podocyte genes contaminated the purified mesangial cells while others did not.

Further analysis of the data indicated that contamination could not explain the observed *Wt1* expression in mesangial cells. Several genes, including *Podxl*, *Nphs1* and *Nphs2* showed strongly podocyte specific expression, as expected, and very low expression levels in mesangial cells. For example, *Nphs2* gave raw signal levels of approximately 5,000 in podocytes, but only about 100 in mesangial cells.

The APBB2 protein, similar to PAWR , was shown to bind to amyloid precursor protein (APP)[19]. It is interesting to note that the gene encoding APP shows strong expression in mesangial cells, with raw signal levels over 4,000, but is also widely and strongly expressed in the other cell types examined, including podocytes and endothelial cells. *Apbb2* has also been shown to be a regulator of programmed cell death [20].

The ARID5B transcription factor has been previously shown to be able to drive smooth muscle development when over expressed in 3T3 fibroblasts [21]. Its strong expression in mesangial cells likely reflects their smooth muscle character. *Arid5b* mutant mice show severe reduction in the number of mesangial cells, as well as their mis-location at the edge of the glomerulus [22]. *Hopx* encodes a divergent homeodomain protein that physically interacts with SRF and regulates gene expression in cardiac muscle [23], and could therefore also play a role in defining the muscle character of mesangial cells.

*Zeb2* encodes a 2-handed zinc finger/homeodomain DNA binding transcriptional repressor that interacts with activated SMADs and represses cadherin expression in response to TGFβ signaling [24]. This is of particular interest since TGFβ is thought to play an essential role in driving kidney fibrosis during a number of diseases, including diabetic nephropathy. Decreased cadherin expression promotes EMT, the production of fibroblasts, and consequent fibrosis. The *Snai2* gene, a member of the snail family, which encodes transcriptional repressors. Notch signaling can activate *Snai2*, promoting resistance to anoikis, and, once again, inducing epithelia to mesenchyme transition through repression of E-cadherin [25].

*Ets1* is most strongly expressed in lymphoid, endothelial and neural crest cells. It plays a key role in cytokine signaling [26]. Its expression in mesangial cells reflects a gene expression signature with significant endothelial character.

The nuclear receptor *Nr2f1* is of interest, as it has been previously shown to have restricted expression in the mesangial cells of the adult kidney, again providing historic validation of the microarray screen.

### Growth factors

Mesangial cells showed very robust expression of three growth factor encoding genes, *Ptn, Mfge8* and *Gdf10*. The *Ptn* gene, encoding pleiotrophin, was expressed at a very high level in mesangial cells, with raw signal values of around 6000. Pleiotrophin is a heparin-binding growth/differentiation inducing cytokine and is a powerful angiogenic factor.

PTN receptors include PTPRB, PTRZ, SDC1 and SDC3. In the prostate PTN has been implicated in the autocrine regulation of mesenchyme and the paracrine regulation of epithelia [27]. We observed modest expression levels of two of these receptor genes (*Ptprb* and *Sdc3*) in mesangial cells, suggesting a possible autocrine role. In addition there was extremely strong expression of *Ptprb* in the glomerular endothelial cells, strongly suggesting a paracrine function. It is also interesting to note that PTN has been shown to disrupt calcium dependent cell-cell adhesion and to promote epithelia to mesenchyme transition [28]. Finally, it has also been observed that PTN has strong antibacterial activity when tested against a panel of gram-positive and gram-negative bacteria [29], arguing that PTN is part of the innate immune system.

*Mfge8* (SED1) has many functions, including promoting the phagocytic removal of apoptotic cells [30]. Pericytes were previously identified as a major source of *Mfge8*[31], and of course mesangial cells can be considered specialized pericytes. In *Mfge8* knockout mice tumor and retinopathy associated angiogenesis was reduced [31]. It is also interesting to note that *Mfge8* has been shown to potentiate PDGFRB signaling [32].

The final robustly expressed growth factor gene with mesangial cell elevated expression was *Gdf10*, which is a member of the *Tgf-β* superfamily and is closely related to *Bmp3*.

### Receptors

The screen of the microarray data revealed a large number of receptor encoding genes with elevated mesangial expression. The top dozen, ranked according to expression levels, were *Itga8*, *Agtr1a*, *Pdgfrb*, *Itga4*, *S1pr3*, *Trpc1*, *Il2rg*, *Ptger4*, *Pla2r1*, *Lphn3*, *P2rx1* and *Tlr4*.

*Itga8*, (*integrin alpha8*) has been previously shown to have glomerular expression that is restricted to the mesangial cells, providing another historical validation of the microarray dataset. The *Agtr1a* gene encodes a receptor for angiotensin, a potent vasopressor hormone. *Agtr1a* mutant mice show hypertrophy of the juxtaglomerular apparatus and mild mesangial expansion [33].

*Pdgfrb* has previously been shown to be expressed in mesangial cells, and the *Pdfrb* mutant mouse shows a failure of mesangial cell development [34]. It is interesting to note that mesangial cells also express *Pdgfc* at robust levels, suggesting a possible autocrine function. *Pdgfc* did not emerge in the screen for mesangial cell enriched growth factors because it is also expressed in other cell types.

The *S1pr3* gene encodes a receptor for sphingosine-1-phosphate. It has been shown to be expressed by macrophages and to play a role in their recruitment during inflammation and atherosclerosis [35]. Reports have also linked *S1pr3* signaling to fibrosis [36].

The *Mrc1* receptor binds the surfaces of pathogenic viruses and bacteria, facilitating their engulfment. It is similar, therefore, in function to the secreted MFGE8, which also promotes phagocytosis. Another receptor associated with a macrophage function is IL2RG, a common subunit for the receptors of a variety of interleukins. In addition the mesangial cells showed a high level of expression of *Tlr4*, which encodes a member of the Toll-like receptors. These are single transmembrane receptors that have a key role in the innate immune system, and are generally found on immune cells. *Tlr4* was previously shown to be expressed by mesangial cells, and to contribute to antibody mediated glomerulonephritis through the production of CXC cytokines [37]. The observed expression of *S1pr3Mrc1Il2rg* and *Mfge8* by mesangial cells serves to better define their anti-bacterial macrophage like character.

Several TRPC receptors were expressed at elevated levels in mesangial cells, including TRPC1, TRPC4 and TRPC6. They belong to the transient receptor potential super family of cation channels that have been implicated in receptor and store-operated calcium entry, and cell death, as well as functioning as sensors for heat, cold, stretch, and osmotic changes.

The P2RX1 receptor functions in mediating synaptic transmission from neurons to smooth muscle, and driving vasoconstriction. The PTERG4 receptor has the opposite effect. It is a receptor for prostaglandin E2 and can induce smooth muscle relaxation.

### *db*/*db* mesangial cells

We next sought to better define the protective and pathogenic gene expression changes that occur in mesangial cells as a function of diabetic nephropathy. We used the *db*/*db* mouse model, which has a mutation in the leptin receptor gene and as a result suffers obesity, diabetes and diabetic nephropathy [38]. We generated *db*/*db* mice that carried the *Meis1-GFP* transgene.

**Figure 4:**
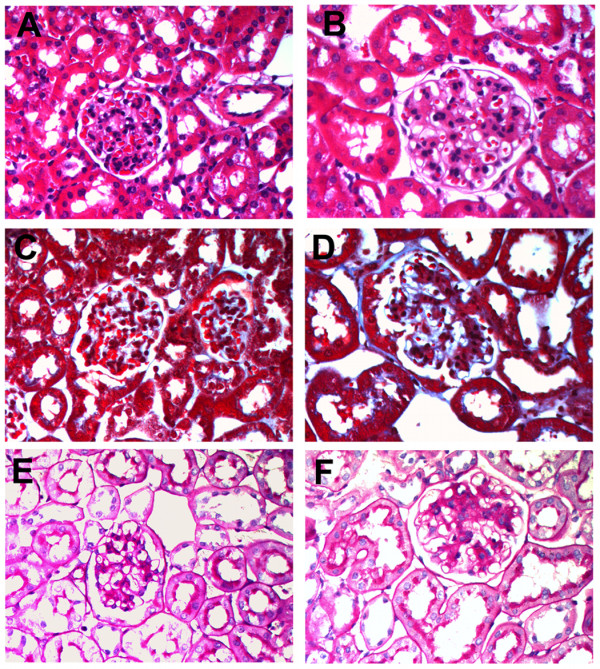
**Glomerular changes in*db/db*diabetic mice.** Panels **A**, **C** and **E** are controls, and panels **B**, **D** and **F** are from *db/db* mice with diabetic nephropathy. Panels **A** and **B** show histological sections stained with hematoxylin and eosin and demonstrate glomeruli hypertrophy. Panels **C** and **D** are sections stained with Masson's trichrome stain. Note the increased staining (blue) indicating a higher level of collagen deposition in the glomeruli of diabetic mice. Panels **E** and **F** are sections stained with Periodic acid-Schiff (PAS) and show mesangial expansion in diabetic mice. All images are representative glomeruli at an original magnification of 200X.

At the age of seven months the *db*/*db* mice showed significant proteinuria, with albumin levels of 994±245 (μg/ml/24hrs), compared to 281±67 for wild type (p=0.0036). The *db*/*db* mice also showed dramatic glycosuria, with glucose levels of 7,580±1,009 mg/dL, compared to 78±6 for wild type. Hematoxylin and eosin staining showed striking glomerular hypertrophy (Figure 4A,B), and Masson’s trichome stain indicated elevated levels of collagen deposition in the glomeruli of *db*/*db* mice (Figure 4C,D). Further, Periodic acid-Shiff (PAS) staining showed clear mesangial expansion in *db*/*db* mice (Figure 4E,F).

**Figure 5:**
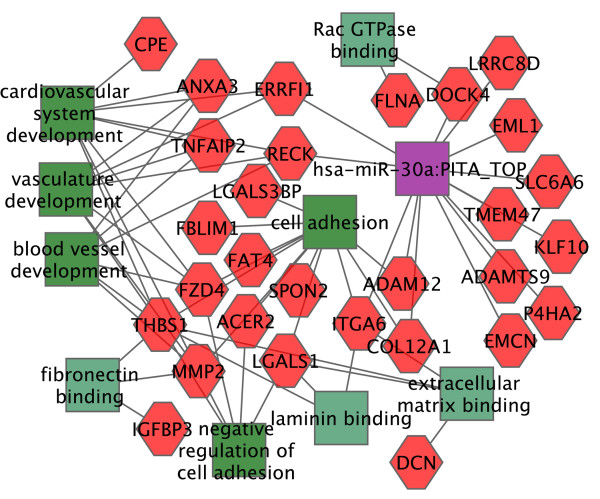
**Changing gene expression programs in mesangial cells of*db/db*diabetic nephropathy mice.** The red hexagons show some of the genes up-regulated in mesangial cells of *db/db* diabetic mice. The dark green squares show biological processes, and light green molecular functions, and purple a microRNA, with lines showing links to related genes.

The mesangial cell gene expression data from biological triplicates of wild type and *db*/*db* mice was analyzed, identifying 184 probesets, with 105 up-regulated and 79 down-regulated in *db*/*db* mesangial cells (Additional file 4: Table S3). We used Toppgene to perform a functional analysis of the list of genes up-regulated (Additional file 5: Table S4). Consistent with the known functions of mesangial cells in the pathogenesis of diabetic nephropathy, the top three molecular functions were extracellular matrix binding, fibronectin binding and laminin binding. Some of the other up-regulated molecular functions of particular interest were Rac GTPase binding, Rho GTPase activator activity, glycoprotein binding, and metallopeptidase activity. The top identified up-regulated biological processes were negative regulation of cell-substrate adhesion, and vascular development. Others included regulation of cell proliferation, which is potentially important in terms of mesangial matrix expansion, immune response, muscle cell differentiation and wound healing. For complete lists see Additional file 5: Table S4. A cytoscape graphics view of some of the up-regulated genes and associated molecular functions, biological processes, and candidate regulating microRNAs is shown in Figure 5.

We then applied a more stringent screen, requiring at least raw signal expression of 300 in three samples, to identify the most robustly expressed up-regulated mesangial cell genes in *db*/*db* mice. Some of the more interesting of the resulting 34 genes are described below.

Thrombospondin (*Tsp1*) showed the greatest fold up-regulation (2.8 fold change) in mice with diabetic nephropathy. TGFβ is primarily secreted as an inactive procytokine that must be activated to bind to its receptors. Thrombospondin plays an important role in this activation process, with *Tsp1* mutant mice showing an inflammatory phenotype similar to that of *Tgf-β* mutant mice [39,40].

TGFβ has been known for many years to play a major role in renal fibrosis. Indeed, it has been argued that most of the features of diabetic nephropathy, including GBM thickening, matrix expansion, hypertrophy and podocyte loss are the result of TGFβ signaling [41]. Further, it has been known for some time that mesangial cells produce thrombospondin [42], and high glucose can up-regulate thrombospondin expression in both rat and human mesangial cells [43]. Elevated TSP1 expression in mesangial cells of human type-2 diabetic nephropathy was previously demonstrated by immunohistochemical staining of renal biopsies [43]. These results provide additional historic validation of the current study. The gene we identified with the greatest up-regulaton, *Tsp1*, has already been shown to play an important role in diabetic nephropathy.

The only collagen gene observed to change significantly in expression was *Col12a1*, which was up-regulated almost two fold in *db*/*db* mice. It is interesting that none of the *Col4* subunit genes showed a change in expression, although their upregulation in diabetic nephropathy has been previously reported [44]. This could be reconciled by a decreased rate of collagen degradation in *db/db* glomeruli [45]. There is also evidence that renal fibroblasts, associated with fibrosis, might be an important source of the increased COL4 [46].

Similarly, there was no increased transcription of PKC genes in the *db/db* mesangial cells, although previous reports have documented increased PKC activation as well as total PKC levels in mesangial cells treated with glucose [47]. Again, the likely explanation is a change in PKC turnover.

*Emcn*, also up-regulated, encodes a mucin like sialoglycoprotein “that interferes with the assembly of focal adhesions complexes and inhibits the interaction between cells and the extracellular matrix” (EMCN Gene Cards) [48]. Other up-regulated genes associated with cell adhesion and the extracellular matrix included *Mmp2* (matrix metallopeptidase 2), and *Lgals1* (lectin, galactose binding). Other ECM related up-regulated genes, but with raw expression levels below 300, included *Fat4Fblim1Spon2Lgals3bpAdam12*, and *Itga6*.

The annexin ANXA3 was also up regulated in *db*/*db* mesangial cells. ANXA3 has been linked to phagocytosis [49], the promotion of angiogenesis [50], the inhibition of phospholipase A2 and anti-coagulation. *Ctsl* encodes cathepsin L, a lysosomal proteinase that has been shown to play a major role in neovascularization.

We also observed up-regulation of ACER2, a ceramidase that cleaves ceramides to generate sphingosine, which has the opposite effects on cell proliferation as survival as sphingosine-1-phosphate. The up-regulated *Ppap2a* gene, encoding phosphatidic acid phosphatase, can dephosphorylate bioactive glycerolipids as well as sphingolipids.

Elevated *Acta2* expression, encoding smooth muscle actin, has been noted previously in diabetic nephropathy mesangial cells, and has been associated with differentiation to a myofibroblast phenotype, believed causally related to fibrosis. Of interest, TGFβ has been shown to induce *Acta2* in mesangial cells grown in culture [51]. We also observed up regulation of *Flna*, encoding filamin A, alpha, which is an actin binding protein involved in cytoskeleletal remodeling to effect changes in cell shape and migration. Another biologically related up-regulated gene was *Wdr1*, which induces the disassembly of actin filaments.

Of particular interest, the Decorin gene, *Dcn*, showed up-regulation in *db*/*db* mesangial cells. Decorin has been shown to interact with many extracellular matrix molecules, including collagen, fibronectin and thrombospondin, as well as the growth factor TGFβ [52]. Expression of *Dcn* has been shown to reduce TGFβ induced fibrosis in many model systems [53]. Indeed, it has been shown that decorin deficiency results in a much more severe diabetic nephropathy in mice with with streptozotocin induced diabetes [54]. This strongly suggests that the over expression of *Dcn* observed in *db*/*db* mesangial cells serves a protective function.

### Validation

**Figure 6:**
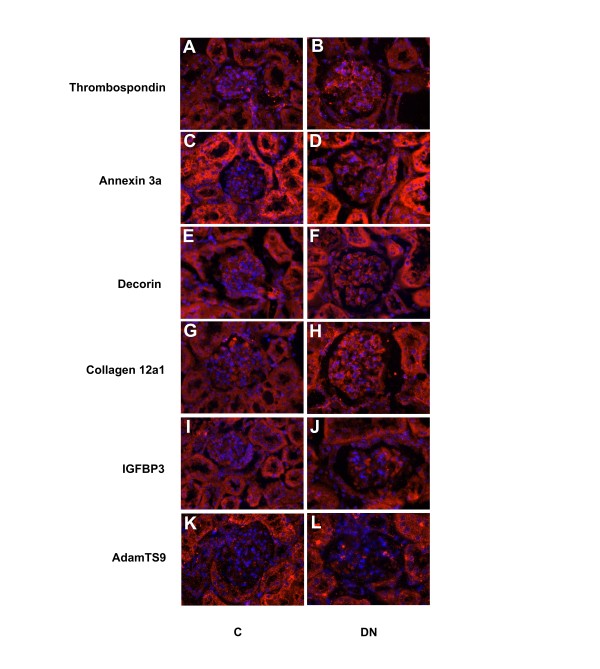
**Immunostaining for validation of gene expression differences in*db/db*glomeruli.** Protein levels were examined in C (control) kidneys, panels **A**, **C**, **E**, **G**, **I** and **K**, as well as DN (diabetic nephropathy) kidneys, panels **B**, **D**, **F**, **H**, **J** and **L**. Elevated glomerular expression was observed in each case, as predicted by the microarray results. In addition, note the increased Collagen12a1 expression in the *db*/*db* tubules (panels **G** and **H**).

To confirm the microarray predicted expression changes we performed immunohistochemisty, comparing wild type glomeruli to those of *db*/*db* mice (Figure 6). The results showed elevated *db*/*db* glomerular expression of Thrombospondin, Annexin3a, Decorin, Collagen12a1, Igfbp3 and AdamTS9, consistent with the microarray results. As might be expected, there were typically restricted regions of over expression. This facilitated detection, since the overall fold change in expression compared to wild type was generally relatively small. Of interest, these genes also showed expression in flanking tubules, which was generally unchanged in the *db*/*db* mouse, although increased somewhat for Decorin and Collagen 12a1.

## Conclusions

In conclusion, we have FACS purified mesangial cells from both normal and diabetic nephropathy model mice and defined their *in vivo* gene expression states. The results contribute to our molecular understanding of the multifunctional character of mesangial cells, with their gene expression signatures showing similarities to pericytes, muscle cells, endothelial cells, neurons, and phagocytes. The expression levels of all growth factor, receptor and transcription factor genes were defined. In addition we analyzed the changes in mesangial gene expression occurring as a function of diabetic nephropathy, identifying genes involved in extracellular matrix, cell division, vasculogenesis, and growth factor modulation. New gene expression changes and biological pathways were discovered, yielding a deeper understanding of the disease process, and candidate targets for the development of novel therapies.

## Competing interests

The authors declare no conflicts of interest.

## Authors’ contributions

E.W.B. performed the experiments and generated the data. S.S.P. and E.W.B. analyzed the data. S.S.P. wrote the manuscript and E.W.B. reviewed/edited the manuscript. Both authors read and approved the final manuscript.

## Pre-publication history

The pre-publication history for this paper can be accessed here:

http://www.biomedcentral.com/1471-2369/13/70/prepub

## Supplementary Material

Additional file 1 **Table S1.** List of 172 genes with elevated expression in normal mesangial cells compared to other renal compartments. Fold change shown is relative to renal vesicles. Click here for file

Additional file 2 **Figure S1.** Heatmap showing genes with elevated expression in normal mesangial cells. Expression patterns of 172 genes with up-regulation in mesangial cells are compared across multiple renal compartments, including cap mesenchyme, renal vesicle, total renal cortex, podocytes, endothelial cells and renal capsule. Red indicates high, yellow represents intermediate and blue indicates low gene expression level. This figure can be zoomed to visualize individual gene names. Click here for file

Additional file 3 **Table S2.** Functional analysis of genes with elevated expression in normal mesangial cells. The top molecular functions, biological processes, cellular components, evolutionarily conserved transcription factor binding sites, microRNAs and diseases associated with the list of 172 genes showing elevated mesangial cell expression. For example, the first molecular function is store-operated calcium channel activity. There are seven genes associated with this function and three of them (*Trpc1*, *Trpc4* and *Trpc6*) show elevated expression in mesangial cells, giving an uncorrected P-value of 0.000017. Functional analysis was carried out with the ToppGene (http://toppgene.cchmc.org/) web tool. Click here for file

Additional file 4 **Table S3.**Genes with altered expression in diabetic nephropathy mesangial cells. The left column lists the Affymetrix probeset IDs. For some genes there are multiple probesets. Column B shows fold change, comparing wild type to db/db mutant. Column C shows directionality of change, with down signifying lower in wild type. The gene with the greatest fold change was Thrombospondin, which was 2.8 fold down in wild type compared to mutant. Click here for file

Additional file 5 **Table S4.** Functional analysis of genes up-regulated in *db/db* diabetic nephropathy mesangial cells. The top molecular function, biological processes, cellular components, evolutionarily conserved transcription factor binding sites and diseases associated with the genes showing up-regulation in *db*/*db* mesangial cells, as determined with the ToppGene (http://toppgene.cchmc.org/) web tool. Click here for file
