# Supplementary figures and images for: Changes in the gene expression programs of renal mesangial cells during diabetic nephropathy
Source: BMC Nephrol. 2012 Jul 28;13:70. doi: 10.1186/1471-2369-13-70 (PMC3416581; doi:10.1186/1471-2369-13-70)

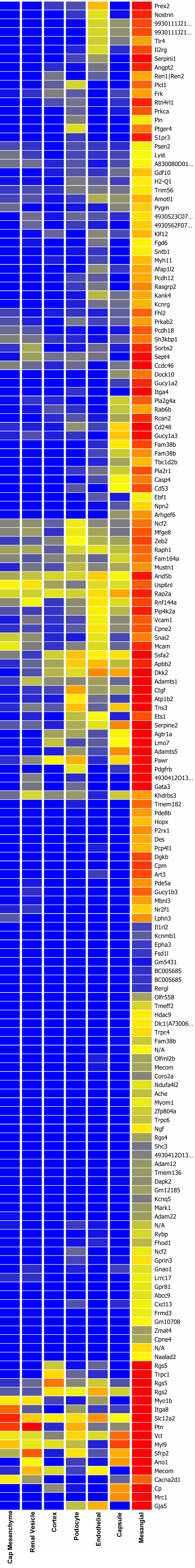

Supplement: Additional file 2 — Figure S1. Heatmap showing genes with elevated expression in normal mesangial cells. Expression patterns of 172 genes with up-regulation in mesangial cells are compared across multiple renal compartments, including cap mesenchyme, renal vesicle, total renal cortex, podocytes, endothelial cells and renal capsule. Red indicates high, yellow represents intermediate and blue indicates low gene expression level. This figure can be zoomed to visualize individual gene names. [file 1471-2369-13-70-S2.tiff]
